# Global topographic uplift has elevated speciation in mammals and birds over the last 3 million years

**DOI:** 10.1038/s41559-021-01545-6

**Published:** 2021-09-02

**Authors:** Javier Igea, Andrew J. Tanentzap

**Affiliations:** Ecosystems and Global Change Group, Department of Plant Sciences, University of Cambridge, Cambridge, UK

**Keywords:** Speciation, Biodiversity, Palaeoecology

## Abstract

Topographic change shapes the evolution of biodiversity by influencing both habitat connectivity and habitat diversity as well as abiotic factors like climate. However, its role in creating global biodiversity gradients remains poorly characterized because geology, climate and evolutionary data have rarely been integrated across concordant timescales. Here we show that topographic uplift over the last 3 million years explains more spatial variation in the speciation of all mammals and birds than do the direct effects of palaeoclimate change and both present-day elevation and present-day temperature. By contrast, the effects of topographic changes are much smaller than those of present-day temperatures in eroded areas. Together, our results stress that historical geological processes rather than traditionally studied macroecological gradients may ultimately generate much of the world’s biodiversity. More broadly, as the Earth’s surface continues to rise and fall, topography will remain an important driver of evolutionary change and novelty.

## Abstract (web summary)

Geology and climate affect speciation. A combination of path analysis applied to palaeo-reconstructions of mammals and birds with analysis of palaeoclimatic data shows that uplift over the last 3 million years explains more spatial variation in speciation than the direct effects of palaeoclimate change or present-day elevation and temperature.

## Main

Understanding how abiotic processes influence the evolution of life on Earth has long fascinated scientists^1^. Integrating historical and present-day data on biodiversity and geology is necessary to determine the drivers of new species formation but has only been possible recently with the advent of more quantitative palaeo-reconstructions^2^. Topographic changes directly promote the formation of new species by shaping the connectivity and environmental conditions of the Earth’s surface^3^. First, uplift and erosion of land can create barriers to dispersal that increase the chances of reproductive isolation and ultimately allopatric speciation^4^. This process has been invoked to explain the high percentage of plant endemism in the world’s mountains^5^. Second, topographic change can create novel habitats and niches where new species evolve and diversify^6^. For example, ecological opportunity linked to the Andean uplift has been shown to drive the fast radiation of endemic lupines^7^. Together, these two mechanisms may explain why global species richness increases with topographic relief^8^ and why high-elevation bird lineages speciate at faster rates than low-lying relatives^9^.

Topographic changes also indirectly promote speciation because they modify climate, which itself affects speciation in at least four ways. First, warmer temperatures at lower elevations may increase mutation rates, either directly through oxygen radical production^10^ or faster metabolic rates that increase DNA synthesis^11^, and shorten generation times, thereby increasing the likelihood of speciation^12,13^ (but see other studies^14,15^). Although endotherms may escape these effects by buffering their body temperatures^16^, warmer temperatures will reduce the costs of maintaining metabolic rates^17^. Lower thermoregulatory costs can release energy that enables large population sizes and promotes speciation by allowing more diverse ecological niches to be occupied^18,19^. Second, species interactions such as competition, predation, and mutualism are a constant source of macroevolutionary change—so called Red Queen effects—and these are expected to be more intense at warmer temperatures^20^. Third, species tend to have wider thermal niches in colder climates because they experience greater temperature variation^21^. This broader niche can reduce opportunities for reproductive isolation and thus speciation if populations are consequently more widespread^20,22^. There may also be greater physiological costs of wider temperature adaptation that restricts the number of species that can use this strategy^18^. Finally, greater climatic instability arising from topographic change may have promoted rapid and repeated ecological speciation by creating novel niches and increased ecological opportunity^16,23^. This relationship between climatic instability and speciation may, however, be non-linear, as higher speciation rates have also been linked with areas of high climatic stability like Pleistocene refugia^24^. Nevertheless, the complex interactions between climate and geology have not to our knowledge been explicitly modelled when attempting to explain patterns of biodiversity at large scales.

Finally, geological history can promote speciation because it influences present-day abiotic factors like elevation and climate. Speciation may be favoured in areas with high present-day elevation, such as if topographic relief increases the range of available niches for species to fill^6^, in addition to any effects from the large historical changes in topography. Thus, the effect of elevational change on both historic and present-day conditions must be considered to quantify accurately which mechanisms are more important drivers of speciation. However, so far, large-scale studies have only correlated present-day elevation with species richness^8^ and speciation rates^9^, without testing direct causal links between topographical change and speciation. To our knowledge, these causal links have only been established for particular mountain ranges^25^ or small groups of species^26,27^, with no large-scale synthesis.

**Fig. 1:**
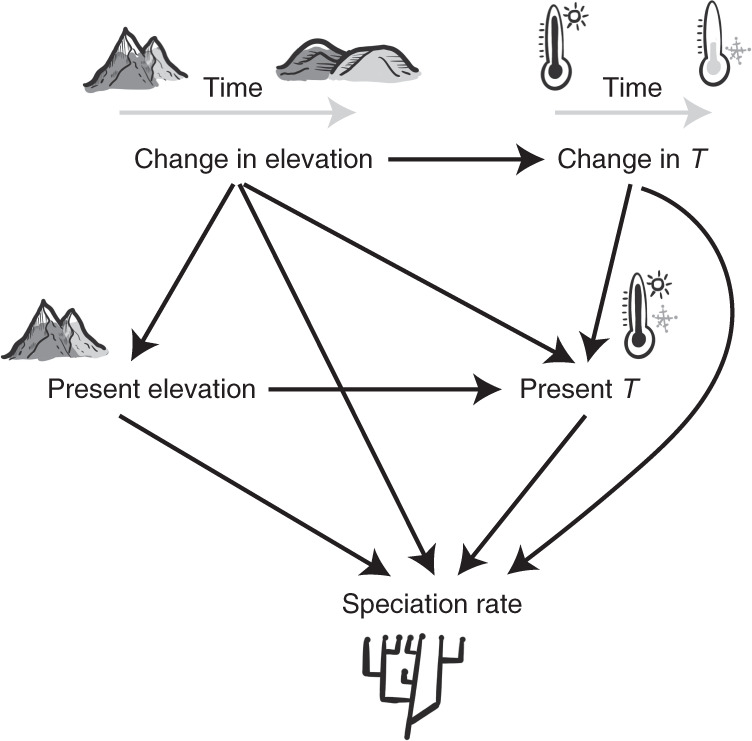
Hypothesized framework of cause and effect between speciation rates and both climatic and geological variables. We considered change in elevation and temperature to be the difference between present-day and mid-Piacenzian (around 3 Ma) values. *T*, annual mean temperature.

**Extended Data Fig. 1:**
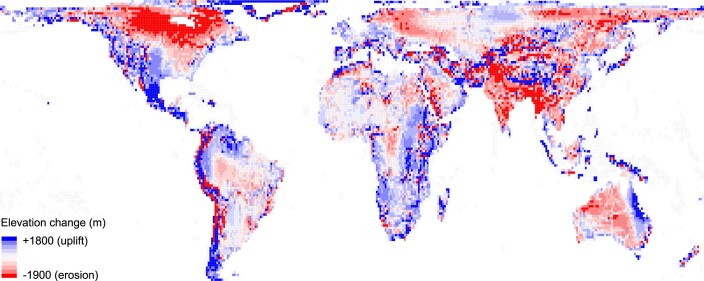
Global elevation changes since the mid-Piacenzian. Pixels are at a 100 km × 100 km resolution and were calculated by subtracting past elevation in the mid-Piacenzian (~3 Ma) from the PRISM4 reconstruction from the ETOPO1 present-day elevation raster.

Here we quantified how much of the present-day spatial variation in speciation rates of mammals and birds was explained by climate and geology and their changes since the Plio-Pleistocene approximately 3 million years ago (Ma). By integrating palaeo-reconstructions in a path analysis framework, we (i) estimated the direct and indirect effects of historical changes in elevation on speciation rates; and (ii) compared these historical effects on speciation rates to those of present-day elevation (Fig. 1). Analysing palaeoclimatic data allowed us to quantify how much of the effect of geology on speciation rates was mediated by climate, namely temperature. As elevation changes can arise from both the gain and the loss of topography, which we respectively termed uplift and erosion, we determined whether these two opposing processes had contrasting effects on speciation. The relatively fine spatial resolution (100 × 100 km) of the palaeo-relief model^28^ allowed us to model changes worldwide rather than only in mountains as considered previously^8,9^ (Extended Data Fig. 1).

## Results

**Fig. 2:**
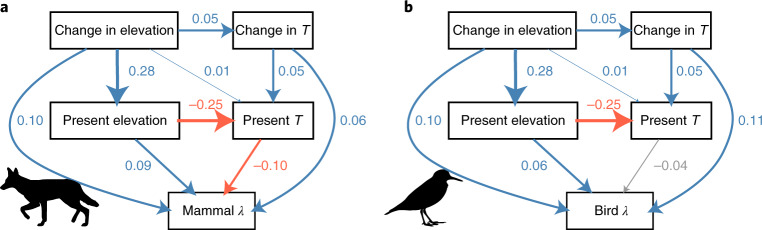
Path analysis of hypothetical causal links among geological and climatic factors and worldwide grid-based speciation rates in uplifted cells. **a**,**b**, Uplifted cells refers to those with an increase in elevation since the Plio-Pleistocene, for mammals (**a**) and birds (**b**). Positive, negative and non-statistically significant (*P* > 0.05) relationships are indicated by blue, red and grey arrows, respectively. The width of the arrows is proportional to the standardized effect size shown by adjacent numbers. Confidence intervals are given in Extended Data Fig. 2. *T*, temperature. Speciation rate (*λ*) was estimated as the weighted mean in each of *n* = 3,780 grid cells as described previously^47^.

**Fig. 3:**
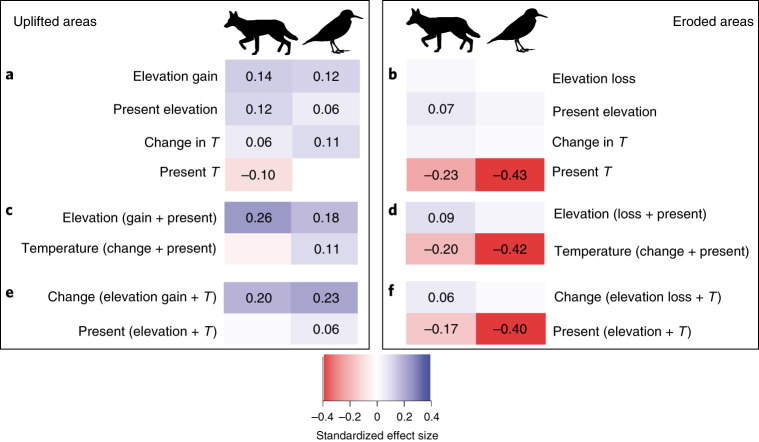
Total (direct and indirect) effects from path analysis of elevation and temperature variables on speciation rates for uplifted and eroded cells. **a**–**f**, Effects on speciation rates for uplifted (**a**,**c**,**e**; *n* = 3,780) and eroded (**b**,**d**,**f**; *n* *=* 10,884) cells in mammals and birds. Effects were estimated for separate predictors of both change and present-day values in each of elevation and temperature (**a**,**b**); all predictors related to elevation and temperature (**c**,**d**); and all predictors related to historical changes and present-day values of a variable (**e**,**f**). Values are standardized effect sizes and are only shown where they cause at least a 5% change in a response for each standard deviation change in the corresponding predictor. *T*, temperature. Speciation rate was estimated as in Fig. 2.

We found that speciation rates increased most in areas with the greatest increases in elevation from the Plio-Pleistocene (Fig. 2). We separately analysed 3,780 and 10,884 100-km grid cells that underwent uplift and erosion, respectively, since the mid-Piacenzian. The positive effect of elevation gain on speciation rates was stronger than that of present-day elevation and outweighed the effects of both temperature in the present day and its historical change (Fig. 3a). Consequently, speciation rates increased by a mean of 11% (95% confidence interval (CI): 9 to 13%) and 10% (95% CI: 8 to 12%) in mammals and birds, respectively, for a 1 standard deviation (s.d.) gain in elevation of 195 m above the mean change in uplift cells of 144 m. Both the direct and the indirect effects of elevation change contributed to these results. Greater historical uplift strongly influenced present-day elevation and subsequently temperature and speciation (Fig. 2). Overall, in uplift areas, the total effects of elevation on speciation rates were larger than the effects of temperature when aggregating both these direct and indirect effects (Fig. 3c). Historical changes in temperature and elevation were also much stronger drivers of speciation in uplifted areas than the present-day values of these variables (Fig. 3e).

**Extended Data Fig. 3:**
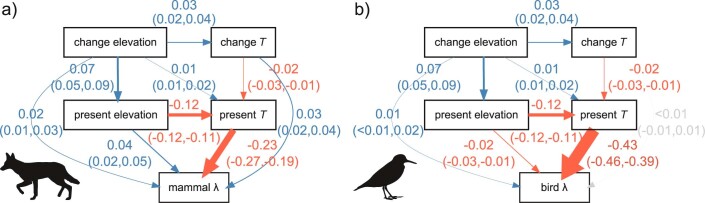
Path analysis of hypothetical causal links among geological and climatic factors and grid-based speciation rates in eroded cells. Uplifted cells were those with a decrease in elevation since the Plio-Pleistocene (n = 10,884 cells) and we fitted a separate path analysis for a) mammals and b) birds. Parentheses show the lower and upper level 95% CI. Colours and lines as in Fig. 2.

Erosion had contrasting effects to those of uplift. Although elevation change causing erosion promoted speciation (Extended Data Fig. 3), it was directly correlated with 5.5- and 11.9-times lower speciation rates than uplift for mammals and birds, respectively (Fig. 3a,b). For example, a 1 s.d. reduction in elevation beneath the mean temporal change of −115 m in eroded cells increased speciation only by a mean of 2% (95% CI: 1 to 3%) and 1% (95% CI: less than 1% to 2%) in mammals and birds, respectively. Instead, temperature—primarily present-day values—had the strongest effect on speciation rates in eroded grid cells (Fig. 3b,d). Historical changes of both elevation and temperature had weaker effects than present-day values (Fig. 3f).

**Fig. 4:**
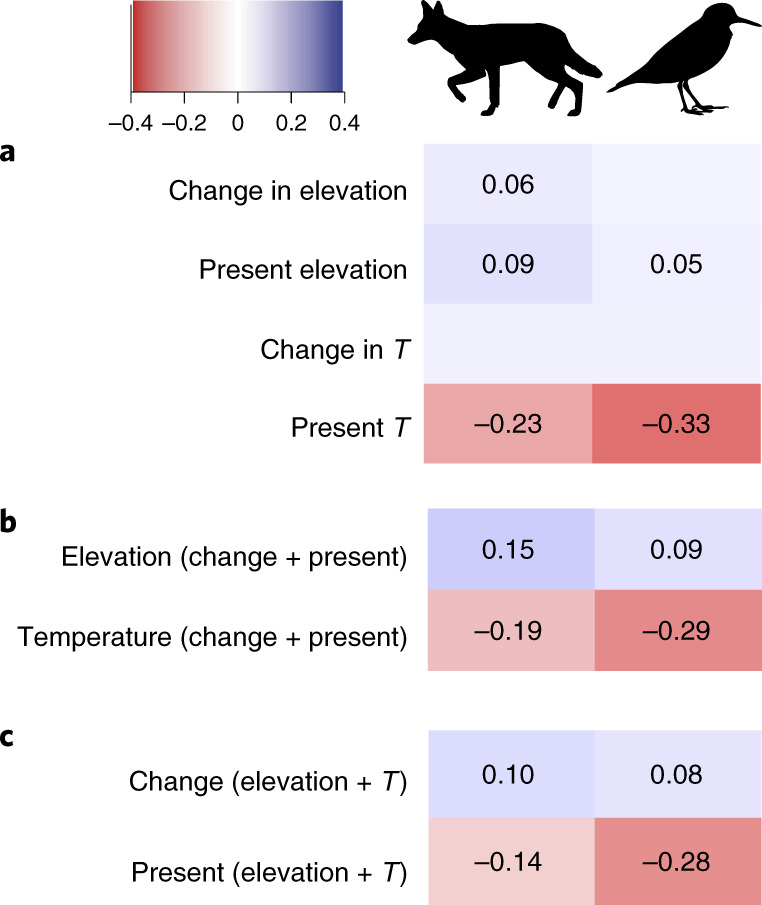
Total (summed direct and indirect) effects from path analysis of elevation and temperature variables on grid-based speciation rates in mammals and birds. **a**–**c**, Effects were calculated for the four predictors in Fig. 2 (**a**); all predictors related to elevation and to temperature (**b**); and all predictors related to historical changes and present-day values of a variable (**c**). *T*, temperature. Speciation rate was estimated as in Fig. 2 and effect sizes are shown only where they change responses by at least 5% as in Fig. 3.

**Extended Data Fig. 4:**
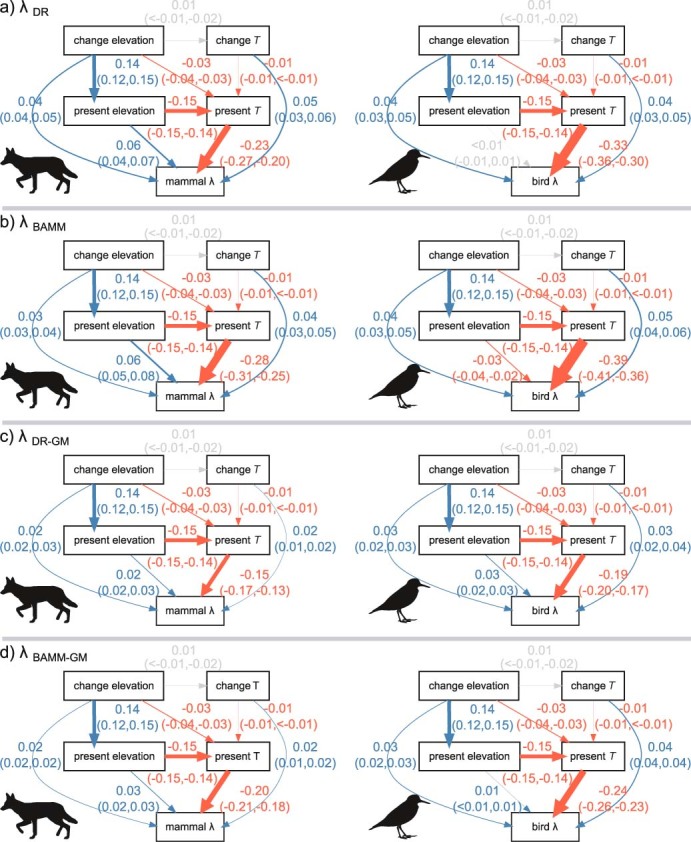
Path analysis of hypothetical causal links among geological and climatic factors and different worldwide grid-based speciation rates for mammals and birds. Models were fitted to **a)** weighted mean *λ*_DR_; **b)** weighted mean *λ*_BAMM_; **c)** geometric weighted mean *λ*_DR - GM_; and **d)** geometric weighted mean *λ*_DR - BAMM_ in each of N = 14,664 cells. Parentheses show the lower and upper level 95% confidence intervals. Lines and colours as in Fig. 2.

The strong effect of topographic change on speciation rates diminished when we analysed all areas together irrespective of whether they underwent uplift or erosion, highlighting the importance of separating these processes (Fig. 4a). In mammals, higher present-day elevation and greater elevation change were similarly directly associated with faster speciation rates (Extended Data Fig. 4). Only elevation changes and not present-day values were directly positively associated with speciation in birds (Extended Data Fig. 4). Elevation overall (that is, when aggregating the effects of both present-day and historical changes) explained less variation in speciation rates in birds than temperature. In mammals, these overall effects were similar (Fig. 4b). This result in mammals may have been partly explained by opposing effects on speciation rates cancelling each other out, as we found positive and negative effects of historical changes and present-day temperature, respectively (Fig. 4b). The effect of the historical changes and the present-day values of the abiotic variables were also comparable in mammals, with the latter stronger predictors in birds (Fig. 4c).

**Extended Data Fig. 5:**
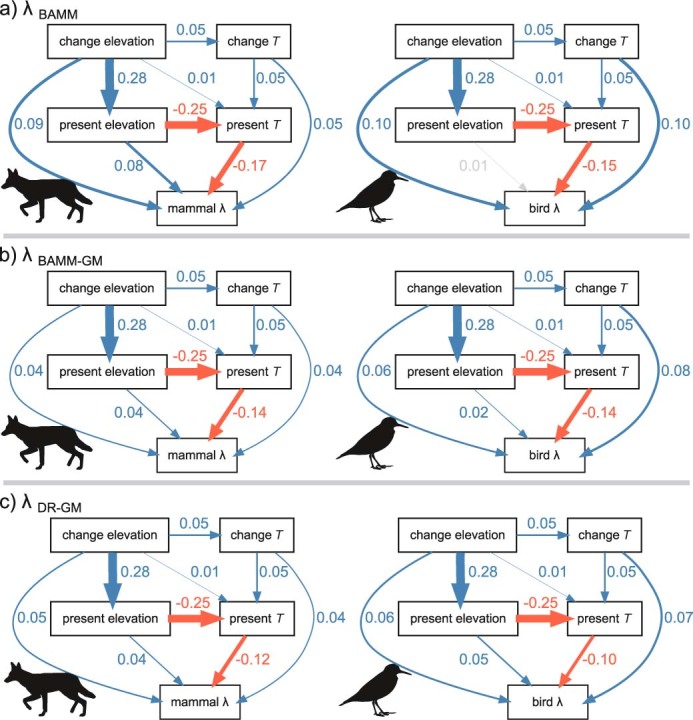
Path analysis of hypothetical causal links among geological and climatic factors and different grid-based speciation rates in uplifted cells. Uplifted cells were those with an increase in elevation since the Plio-Pleistocene (n = 3,780 cells) and we fitted a separate path analysis for mammals and birds. Models were fitted to **a)** weighted mean *λ*_BAMM_; **b)** geometric weighted mean *λ*_BAMM - GM_; and **c)** geometric weighted mean *λ*_DR - GM_. Lines and colours as in Fig. 2.

**Extended Data Fig. 6:**
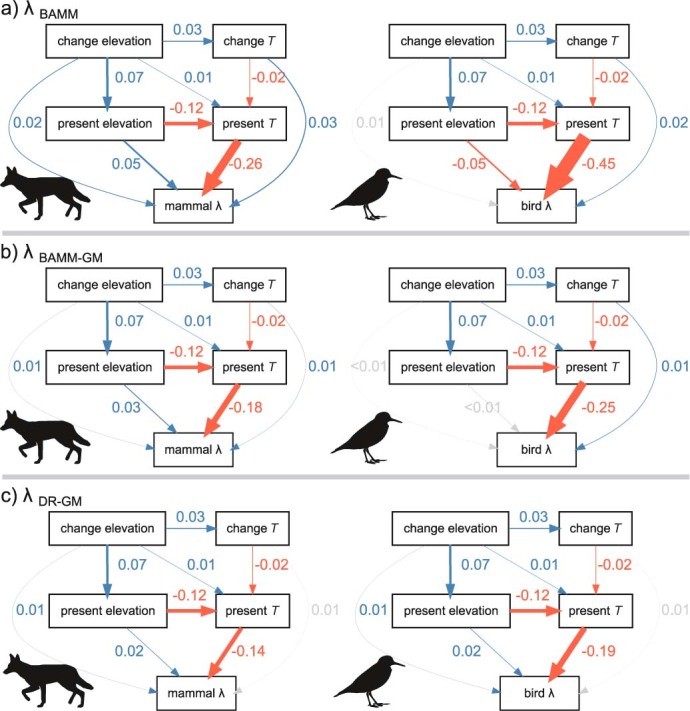
Path analysis of hypothetical causal links among geological and climatic factors and different grid-based speciation rates in eroded cells. Eroded cells were those with a decrease in elevation since the Plio-Pleistocene (n = 10,884 cells) and we fitted a separate path analysis for mammals and birds. Models were fitted to **a)** weighted mean *λ*_BAMM_; **b)** geometric weighted mean *λ*_BAMM - GM_; and **c)** geometric weighted mean *λ*_DR - GM_. Lines and colours as in Fig. 2.

**Extended Data Fig. 7:**
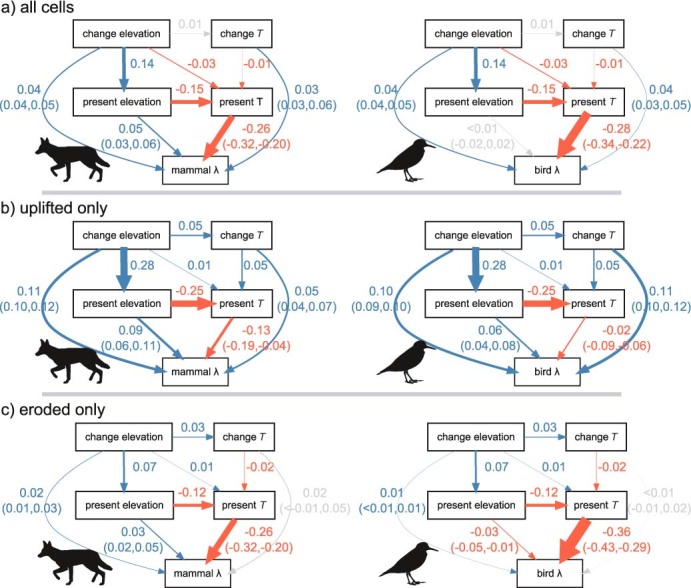
Path analyses of hypothetical causal links among geological and climatic factors and grid-based speciation rates using 100 random trees in mammals and birds. Models were fitted to **a)** all cells (n = 14,664); **b)** only uplifted cells (n = 3,780); and **c)** only eroded cells (n = 10,884 cells). Median values of effect sizes are shown with the lower and upper level 95% CI boundaries in parentheses. Lines and colours as in Fig. 2.

**Extended Data Fig. 8:**
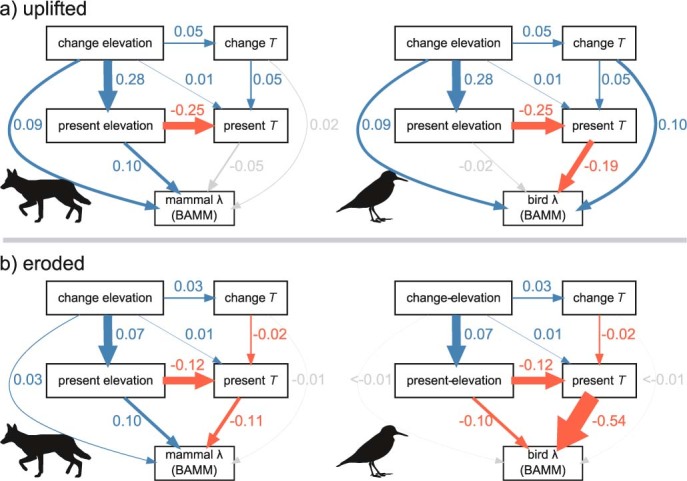
Path analysis of hypothetical causal links among geological and climatic factors and grid-based speciation rates over the last 3 million years. There were 762 and 1923 species of mammals and birds with an age <3 Myr, respectively. Including only these species in our analyses resulted in excluding 363 and 242 cells where all mammals and bird species were older, respectively. We then calculated the weighted mean *λ*_BAMM_ in a) uplifted (n = 3719 cells) and b) eroded (n = 10,528 cells for mammals and birds, respectively). As in our main analyses, we found that historic changes in elevation had comparable effects on speciation to current elevation (compare panel a) to Extended Data Fig 5a and panel b) to Extended Data Fig 6a). This result was unsurprising given the strong correlation between speciation rates. Weighted mean *λ*_BAMM_ for species younger than 3 Myr was strongly positively correlated with rates calculated for all species reported in the main text (ρ = 0.87 and 0.93 for mammals and birds, respectively, p <0.001 for both. Lines and colours as in Fig. 2.

Our results were robust to alternative metrics of speciation rates and to topological uncertainty in the mammal and bird phylogenies. First, we repeated the path analyses using alternative estimates of spatially explicit speciation rates (see Methods). Mirroring our main results (Figs. 2–4), these alternative analyses showed that uplift and erosion had contrasting effects on speciation rates (Extended Data Figs. 5 and 6). Second, we confirmed that the estimated effect sizes on speciation rates obtained with the maximum clade credibility (MCC) trees for mammals and birds (Figs. 2–4) were within the confidence intervals of the effect sizes obtained when running the analyses with 100 random trees from the posterior distributions of the phylogenetic reconstructions (Extended Data Fig. 7). Finally, our results were unchanged when we repeated our analyses using spatially explicit speciation rates estimated only over the period of our palaeo-elevation data; that is, the last 3 million years (Extended Data Fig. 8).

## Discussion

Here we found that historical elevation uplift explained more spatial variation in speciation of two large vertebrate classes than did the direct effects of present-day environmental conditions. Higher-elevation lineages have been previously shown to speciate at faster rates in global mountains^9^. By incorporating reconstructions of past topography and climate, our findings expand on this association by showing that gain in elevation more strongly promotes recent speciation rates than simply the presence of high present-day elevation. More broadly, these results point to the importance of both direct and indirect historical processes for explaining spatial patterns of biodiversity. Although these historical processes have often been appreciated^8^, their importance has not yet been rigorously quantified alongside more traditional macroecological gradients like present-day temperature and elevation.

Geological uplift generally decreases surface temperatures and increases the rate of temperature change^29,30^, but this relationship has rarely been explicitly modelled when quantifying the role of abiotic factors in speciation. Higher rates of past temperature change have been shown to be associated with increased speciation rates and species turnover in mountains^9^. For example, colonizations and radiations during the Pleistocene interglacial and glacial periods are one of the main drivers of recent speciation in temperate regions^31,32^. By using path analyses, we expanded on these results and estimated that the direct contributions of changes in elevation and temperature on speciation were comparable at a global scale.

Our analyses also revealed that opposing geological forces like surface uplift and erosion have contrasting effects on biodiversity. In areas where elevation increased from the Pleistocene, this gain in elevation had the largest positive effect on speciation rates (Fig. 3a). This result may arise because areas with active uplift have increased habitat complexity, new niches and biogeographic barriers, all of which can increase reproductive isolation and ultimately cause speciation^33,34^. By contrast, in areas where elevation decreased from the Pleistocene, the effect of geological change on speciation was much smaller (Fig. 3b). These regions where erosion was large may have lost habitat diversity and experienced greater extinction rates; for example, analogous to the maturity phase on oceanic islands^35^. Temperature may therefore remain a stronger predictor of speciation rates than habitat diversity in these eroded areas.

Mammals and birds responded somewhat differently to the abiotic gradients we studied, which potentially reflects contrasting ecological strategies. The effects of present-day elevation were stronger in mammals than in birds, which were themselves more strongly affected by current temperature (Fig. 2). Elevation creates physical barriers to dispersal, contributing to smaller ranges in mammals^36^. The resulting reductions in gene flow may mean that geographic isolation is more easily associated with speciation in mammals than in birds^37^. By contrast, speciation may be more strongly associated with temperature in birds because variation in temperature creates larger differences in the timing and extent of mating than in mammals^38^, and thus more chances for reproductive isolation. Future work should test how the effects of geological processes on biogeography vary with the life history and dispersal strategies of organisms. Exploring whether speciation rates vary across communities with contrasting morphospaces would also elucidate the role of ecological strategies in speciation driven by environmental change.

Integrating climatic, geological and biological datasets is essential to untangle the mechanisms that generate life on Earth^2^. Our study used an integrative approach of this kind to uncover and quantify the role of geological changes as drivers of biodiversity at notable taxonomic and geographic scales. One limitation is that we were unable to estimate the uncertainty related to the palaeo-reconstructions, as quantitative estimates do not yet exist. As novel palaeo-altimetric methods are further refined and more precise reconstructions of past elevation are generated^39,40^, the association between speciation and geological changes can be improved and assessed in older epochs than the Plio-Pleistocene. Only through these joint analyses of biological processes and their surrounding abiotic factors, as Von Humboldt proposed^1^, can we begin to comprehend the mechanisms that generate new species.

## Methods

### Phylogenies and species distribution data

We analysed widely used and near-complete phylogenies accounting for phylogenetic uncertainty. For terrestrial mammals, we first obtained 100 random trees from the pseudoposterior provided previously^41^. Following a previous study^42^, we recalibrated these 100 trees with the dates from another study^43^ using PATHd8^44^. We then used the recalibrated 100 trees to obtain an MCC tree using TreeAnnotator v.1.8.2^45^. For birds, we estimated the MCC Tree with phangorn^46^ using 100 random trees from the updated version of the posterior distribution in another study^47^. As all downstream results were virtually identical between the MCC trees and the 100 random trees (Extended Data Fig. 7), we present only the former in the main text.

Distribution data were obtained from the International Union for the Conservation of Nature (IUCN) Red List for mammals (v.5.2) and from BirdLife International (v.6.0) for birds. We collated the phylogenetic and distribution data by standardizing the species names using the IUCN and BirdLife taxonomies to obtain a final dataset of 4,633 and 9,622 species of mammals and birds, respectively.

### Speciation rate estimates

We estimated two species-specific speciation rates (*λ*) in two different ways using the mammal and bird phylogenies. First, we calculated the diversification rate (DR) statistic (herein, *λ*_DR_)^47^. This metric is calculated for each species as the inverse of the equal splits measure; that is, the sum of the branch lengths separating a tip from the root, in which each successive branch from the tip is multiplied by a factor of 0.5 to give greater weight to branches closer to the present^48^. Second, we used Bayesian analysis of macroevolutionary mixtures (BAMM)^49^ to obtain tip estimates of speciation rates (herein, *λ*_BAMM_) while accounting for rate heterogeneity both across lineages and through time and accommodating for non-random taxon sampling. Sampling fractions for BAMM were implemented at the family level by dividing the number of species present in the tree in a family by the number of species described in that family according to the IUCN and BirdLife taxonomies (see above). Each BAMM analysis was run for 50 million generations with priors generated by the function “setBAMMpriors” in the R package BAMMtools^49^ and the “expectedNumberOfShifts” parameter was set to 50. We discarded the initial 5 million generations as burn-in and we confirmed that the effective sample sizes for the log-likelihood and the number of rate shifts were above 200. Although the reliability of BAMM estimates has been questioned^50,51^, simulations show that robust estimates of *λ* can be estimated with large datasets like ours^52,53^.

Both of our speciation rates were conditioned on evolutionary history in the recent past. Specifically, rates estimated along the terminal branches of phylogenetic trees represent the waiting time in the present day before a lineage will undergo another speciation event^54^. These estimates of present-day speciation rates can be robustly estimated despite considerable uncertainty about diversification histories deeper within phylogenetic trees^55^ and without palaeontological data to inform rate estimation^52,53^. Speciation rates, unlike diversification rates, are also generally robust to the effect of unmeasured extinction events^54^. For all these reasons, present-day speciation rates are widely used to explain spatial patterns of biodiversity^14,15,54^ and we used them in our analyses.

### Spatial variation in speciation rates

We mapped speciation rates by overlaying species ranges with a grid of 100-km by 100-km cells. This approach allowed us to account for variation in local species pools among grid cells. Following past studies^14,47^, we then computed the speciation rate for individual grid cells with at least one mammal and one bird species. We estimated the arithmetic mean of *λ*_BAMM_ and *λ*_DR_ for the species present in each cell but weighted each species by the inverse of their range (that is, the number of grid cells in which they occurred); herein, weighted mean *λ*. Weighting decreases the contribution of widespread species to the overall mean in each cell. Second, we calculated the geometric mean of *λ*_BAMM_ and *λ*_DR_ by weighting species by the inverse of their range (herein, weighted geometric mean *λ*_BAMM/DR_).

### Abiotic gradients

We estimated present-day elevation across the grid cells using a 1-arcmin (around 1.9-km resolution) global relief model from the United States National Oceanic and Atmospheric Administration (ETOPO1^56^). We masked the sea values, re-projected the raster to an equal-areas projection, and overlaid it onto the 100 × 100-km grid of cells used for speciation estimates. Then, we computed mean values of elevation for each cell in the grid. Past elevation was derived from a 1-degree (around 111-km resolution) global reconstruction of topography in the mid-Piacenzian (around 3 Ma, PRISM4^28^). As above, sea values were masked and the palaeo-relief model was re-projected before computing mean elevation per cell in the grid. We estimated the historical change in elevation as the difference in mean elevation per cell between the present and the past (Extended Data Fig. 1). Cells in which the mean past elevation was negative, corresponding to 10.7% of the total and almost exclusively within the oceans, were discarded to ensure land masses were present for the entirety of our comparisons.

A 2.5-arcmin (around 4.6-km resolution) global temperature raster was obtained for present-day and past conditions (M2 Late Pliocene, around 3.3 Ma) from WorldClim^57^ and PaleoClim^58^, respectively. As above, rasters were re-projected and overlaid with the grid of speciation estimates to compute the mean present and change in temperature (present minus past) for each cell.

### Statistical analyses

We tested how speciation rates varied with historical changes and present-day elevation and temperature using path analysis. This framework allowed us to incorporate our proposed cause–effect relationships among the predictor variables. Using the R package piecewiseSEM^59^, we modelled the direct and indirect effects on grid-based speciation rates (for example, weighted mean *λ*_DR_) from both present-day elevation and temperature and their historical changes. The indirect effects were mediated by historical changes in elevation and temperature determining the present-day values of each variable, and thus speciation rates. We also assumed that historical changes in elevation could influence historical changes in temperature and that present-day elevation influenced present-day temperatures (Fig. 1). All predictor variables were centred and scaled and both present-day elevation and speciation rates were log-transformed. No collinearity was detected among the predictors (that is, Spearman’s *ρ* was less than 0.6 for all pairwise comparisons). Preliminary analyses revealed significant spatial autocorrelation in the residuals of the linear regressions (Moran’s *I* *λ*_DRmammals_ = 0.479, *P* < 0.001; Moran’s *I* *λ*_DRbirds_ = 0.568, *P* < 0.001), so we implemented spatial simultaneous autoregressive (SAR) error models with the R package spdep^60^. A test of missing paths in our model also identified a need to include a link from historical changes in elevation to present-day temperature (Fig. 1). We did not include any further missing paths that linked present-day conditions to historical changes, which would have created bidirectional relationships in our model. Total effects on speciation rates for each predictor were calculated by adding the direct and indirect effects.

### Reporting Summary

Further information on research design is available in the Nature Research Reporting Summary linked to this article.

## Supplementary information

Reporting Summary

Peer Review Information

## Figures and Tables

**Extended Data Fig. 2:**
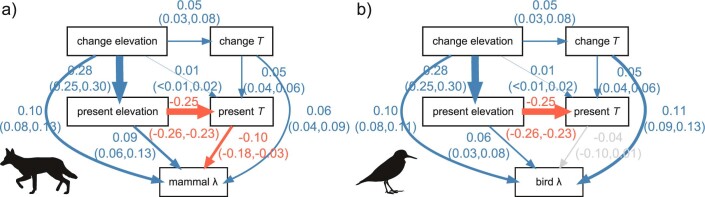
Path analysis of hypothetical causal links among geological and climatic factors and grid-based speciation rates in uplifted cells. Uplifted cells were those with an increase in elevation since the Plio-Pleistocene (n = 3,780 cells) and we fitted a separate path analysis for a) mammals and b) birds. Parentheses show the lower and upper level 95% CI. Colours and lines as in Fig. 2.

## Data Availability

The raw data that support the findings of this study are available from the National Oceanic and Atmospheric Administration for present-day elevation in ETOPO1 with the identifier 10.7289/V5C8276M; the United States Geological Survey for palaeo-elevation in PRISM4 (https://geology.er.usgs.gov/egpsc/prism/4_data.html); WorldClim for present-day temperatures (http://www.worldclim.org); figshare for palaeo-temperatures with the identifier 10.6084/m9.figshare.c.4126292.v1; the IUCN Red List for mammal distributions (http://www.iucnredlist.org); BirdLife for bird species distributions (http://datazone.birdlife.org); the supplementary materials of a previous study^41^ for the mammal phylogeny (10.1111/j.2041-210X.2011.00103.x); and the supplementary data of another previous study^47^ for the bird phylogeny.

## Code availability

All code required to reproduce the analyses from the raw data is available at https://figshare.com/s/977f006d2ed37bd4b4ef.
