# Supplementary material for: Global topographic uplift has elevated speciation in mammals and birds over the last 3 million years
Source: Nat Ecol Evol. 2021 Sep 2;5(11):1530–5. doi: 10.1038/s41559-021-01545-6 (PMC8560637; doi:10.1038/s41559-021-01545-6)
Supplement: Supplementary file 1 — Reporting Summary [file 41559_2021_1545_MOESM1_ESM.pdf]

nature portfolio

Corresponding author(s): Javier Igea
Andrew Tanentzap

Last updated by author(s): Jul 25, 2021

Reporting Summary

Nature Portfolio wishes to improve the reproducibility of the work that we publish. This form provides structure for consistency and transparency in reporting. For further information on Nature Portfolio policies, see our Editorial Policies and the Editorial Policy Checklist.

Statistics

For all statistical analyses, confirm that the following items are present in the figure legend, table legend, main text, or Methods section.

n/a	Confirmed	
☐	☒	The exact sample size (*n*) for each experimental group/condition, given as a discrete number and unit of measurement
☒	☐	A statement on whether measurements were taken from distinct samples or whether the same sample was measured repeatedly
☐	☒	The statistical test(s) used AND whether they are one- or two-sided<br>*Only common tests should be described solely by name; describe more complex techniques in the Methods section.*
☐	☒	A description of all covariates tested
☐	☒	A description of any assumptions or corrections, such as tests of normality and adjustment for multiple comparisons
☐	☒	A full description of the statistical parameters including central tendency (e.g. means) or other basic estimates (e.g. regression coefficient) AND variation (e.g. standard deviation) or associated estimates of uncertainty (e.g. confidence intervals)
☐	☒	For null hypothesis testing, the test statistic (e.g. *F*, *t*, *r*) with confidence intervals, effect sizes, degrees of freedom and *P* value noted<br>*Give P values as exact values whenever suitable.*
☐	☒	For Bayesian analysis, information on the choice of priors and Markov chain Monte Carlo settings
☒	☐	For hierarchical and complex designs, identification of the appropriate level for tests and full reporting of outcomes
☐	☒	Estimates of effect sizes (e.g. Cohen's *d*, Pearson's *r*), indicating how they were calculated

Our web collection on statistics for biologists contains articles on many of the points above.

Software and code

Policy information about availability of computer code

Data collection	n/a
Data analysis	The following software was used to analyse the data in this study: PATHd8, TreeAnnotator v.1.8.2, R v.3.6, R packages phangorn, BAMMtools, piecewisesem, spdep. All code to reproduce the analyses have been deposited in figshare (hyperlink: https://figshare.com/s/977f006d2ed37bd4b4ef).

For manuscripts utilizing custom algorithms or software that are central to the research but not yet described in published literature, software must be made available to editors and reviewers. We strongly encourage code deposition in a community repository (e.g. GitHub). See the Nature Portfolio guidelines for submitting code & software for further information.

Data

Policy information about availability of data

All manuscripts must include a data availability statement. This statement should provide the following information, where applicable:

- Accession codes, unique identifiers, or web links for publicly available datasets
- A description of any restrictions on data availability
- For clinical datasets or third party data, please ensure that the statement adheres to our policy

The data that support the findings of this study are available from NOAA for present-day elevation in ETOPO1 with the identifier doi:10.7289/V5C8276M, USGS for paleo-elevation in PRISM4 (hyperlink: https://geology.er.usgs.gov/egpsc/prism/4_data.html), Worldclim for present-day temperatures (hyperlink: http://www.worldclim.org), figshare for paleo-temperatures with the identifier doi:10.6084/m9.figshare.c.4126292.v1, IUCN Red List for mammal distributions (hyperlink: http://www.iucnredlist.org), Birdlife for bird species distributions (hyperlink: http://datazone.birdlife.org), supplementary materials of Methods in Ecology and

March 2021

Evolution for the mammal phylogeny with the identifier doi: 10.1111/j.2041-210X.2011.00103.x, and supplementary data of Nature for the bird phylogeny with the identifier doi: 10.1038/nature11631.

Field-specific reporting

Please select the one below that is the best fit for your research. If you are not sure, read the appropriate sections before making your selection.

☐ Life sciences ☐ Behavioural & social sciences ☒ Ecological, evolutionary & environmental sciences

For a reference copy of the document with all sections, see nature.com/documents/nr-reporting-summary-flat.pdf

Ecological, evolutionary & environmental sciences study design

All studies must disclose on these points even when the disclosure is negative.

Study description	We quantified how much of the present-day spatial variation in speciation rates of mammals and birds was explained by present-day temperature and elevation and changes in temperature and elevation since the Plio-Pleistocene approximately 3 million years ago.
Research sample	The research was based on existing spatial and phylogenetic data for 4633 and 9622 species of mammals and birds, respectively. This sample was the largest number of species with both distribution and phylogenetic data. Spatial data for mammals and birds can be obtained from the IUCN Red List (http://www.iucnredlist.org, version 5.2) and Birdlife (http://datazone.birdlife.org/home, version 6), respectively. Phylogenetic data for mammals and birds came from doi:10.1111/j.2041-210X.2011.00103.x and doi:10.1038/nature11631, respectively.
Sampling strategy	Sample sizes were chosen to maximize the number of species with both phylogenetic and distributional data.
Data collection	No new data were collected in this study.
Timing and spatial scale	There is no temporal scale corresponding with data collection. All environmental data were taken from a 100x100 km spatial resolution.
Data exclusions	Species without both phylogenetic and distributional data were excluded.
Reproducibility	Analyses were repeated with different ways to measure speciation to verify the reproducibility of the study findings.
Randomization	Randomization was not relevant as no experiments were performed in this study that assigned experimental units to different treatment groups.
Blinding	Blinding was not relevant as no experiments were performed in this study that assigned experimental units or participants to different treatment groups.

Did the study involve field work? ☐ Yes ☒ No

Reporting for specific materials, systems and methods

We require information from authors about some types of materials, experimental systems and methods used in many studies. Here, indicate whether each material, system or method listed is relevant to your study. If you are not sure if a list item applies to your research, read the appropriate section before selecting a response.

Materials & experimental systems

n/a	Involved in the study	
☒	☐	Antibodies
☒	☐	Eukaryotic cell lines
☒	☐	Palaeontology and archaeology
☒	☐	Animals and other organisms
☒	☐	Human research participants
☒	☐	Clinical data
☒	☐	Dual use research of concern

Methods

n/a	Involved in the study	
☒	☐	ChIP-seq
☒	☐	Flow cytometry
☒	☐	MRI-based neuroimaging
