# Supplementary material for: Global topographic uplift has elevated speciation in mammals and birds over the last 3 million years
Source: Nat Ecol Evol. 2021 Sep 2;5(11):1530–5. doi: 10.1038/s41559-021-01545-6 (PMC8560637; doi:10.1038/s41559-021-01545-6)
Supplement: Supplementary file 2 — Peer Review Information [file 41559_2021_1545_MOESM2_ESM.pdf]

natureresearch

Peer Review Information

Journal: Nature Ecology & Evolution
Manuscript Title: Global topographic uplift has elevated speciation in mammals and birds over the last 3 million years
Corresponding author name(s): Javier Igea, Andrew J Tanentzap

Editorial Notes:

**Redactions – published data**	Parts of this Peer Review File have been redacted as indicated to remove third-party material.

Reviewer Comments & Decisions:

Decision Letter, initial version:

15th March 2021

*Please ensure you delete the link to your author homepage in this e-mail if you wish to forward it to your co-authors.

Dear Andrew,

Your Article, "Global topographic uplift has elevated speciation in mammals and birds over the last 3 million years" has now been seen by three reviewers. You will see from their comments copied below that while they find your work of considerable potential interest, they have raised quite substantial concerns that must be addressed. In light of these comments, we cannot accept the manuscript for publication, but would be very interested in considering a revised version that addresses these serious concerns.

We hope you will find the reviewers' comments useful as you decide how to proceed. If you wish to submit a substantially revised manuscript, please bear in mind that we will be reluctant to approach the reviewers again in the absence of major revisions.

If you choose to revise your manuscript taking into account all reviewer and editor comments, please highlight all changes in the manuscript text file in Microsoft Word format.

We are committed to providing a fair and constructive peer-review process. Please do not hesitate to contact us if there are specific requests from the reviewers that you believe are technically impossible or unlikely to yield a meaningful outcome.

If revising your manuscript:

* Include a "Response to reviewers" document detailing, point-by-point, how you addressed each referee comment. If no action was taken to address a point, you must provide a compelling argument. This response will be sent back to the referees along with the revised manuscript.

* If you have not done so already we suggest that you begin to revise your manuscript so that it conforms to our Article format instructions at http://www.nature.com/natecolevol/info/final-submission. Refer also to any guidelines provided in this letter.

* Include a revised version of any required reporting checklist. It will be available to referees (and, potentially, statisticians) to aid in their evaluation if the manuscript goes back for peer review. A revised checklist is essential for re-review of the paper.

Please use the link below to submit a revised paper:

[REDACTED]

Note: This URL links to your confidential home page and associated information about manuscripts you may have submitted, or that you are reviewing for us. If you wish to forward this email to co-authors, please delete the link to your homepage.

If you wish to submit a suitably revised manuscript we would hope to receive it within 6 months. If you cannot send it within this time, please let us know. We will be happy to consider your revision so long as nothing similar has been accepted for publication at Nature Ecology & Evolution or published elsewhere.

Nature Ecology & Evolution is committed to improving transparency in authorship. As part of our efforts in this direction, we are now requesting that all authors identified as 'corresponding author' on published papers create and link their Open Researcher and Contributor Identifier (ORCID) with their account on the Manuscript Tracking System (MTS), prior to acceptance. This applies to primary research papers only. ORCID helps the scientific community achieve unambiguous attribution of all scholarly contributions. You can create and link your ORCID from the home page of the MTS by clicking on 'Modify my Springer Nature account'. For more information please visit please visit www.springernature.com/orcid.

Please do not hesitate to contact me if you have any questions or would like to discuss the required revisions further.

Thank you for the opportunity to review your work.

[REDACTED]

Reviewer expertise:

Reviewer #1: macroecology patterns, including mountain diversity

Reviewer #2: structural equation modeling in ecology

Reviewer #3: macroecology patterns, including mountain diversity

Reviewers' comments:

Reviewer #1 (Remarks to the Author):

This is a reasonably well-written and potentially very interesting analysis that adresses the hypothesis that active dynamics of mountain uplift has driven speciation globally. The key new thing they bring to the table is a map of elevation at high spatial resolution from 3 million years ago, allowing a high-resolution map of which grid cells have been uplifted or eroded away in the time since. This represents a nice resource to test a question that has otherwise been relatively intractable. Somewhat inexplicably, though, that map doesn't appear anywhere in the paper, which I feel rather inhibits the ability to evaluate the analysis.

The authors use path analysis to evaluate the relative contributions of current values of elevation and temperature and the changes in those variables. That seems to me to be a relevant way to do it, and the relationships between the variables is realistically represented. This is one way of addressing the inherent lack of mutual independence in the contrasted predictors.

However, the big problem when doing this type of analysis is that uplift, of course, is associated with mountains, and a host of other mechanisms have associated mountains with increased species richness / speciation, incl climatic dynamics, tight packing of climate types, and complexity of geological substrates. Because I haven't seen the actual uplift map it's hard for me to tell whether their result simply is that you have more speciation in mountains, which is well known.

Also, their method for evaluating specation rates, the DR metric, is not associated with a certain time scale (and has massive problems of phylogenetic autocorrelation which are very hard to deal with - and the same goes for bamm), thought they are explicitly looking at uplift over the last 3 mio years. I would think a metric of speciation rate explicitly in the time period was more relevant.

A theoretical concern is that they use temperature as a predictor of speciation rates, but that does not actually have a clear evolutionary interpretation, as far as I know. They argue that temperature might affect mutation rates, and thus the accumulation of genetic divergence among populations, but that hypothesis explicitly does not concern endotherms, which are the study subjects here.

With regards to their analysis I don't understand why they reapply a 1-degree grid net to a 100x100 km equal area grid. The data is on 1-degree, any reprojection at the same resolution must necessarily throw away great amounts of data. I don't immediately see how different sizes towards the poles of the 1-deg should be a problem for analyzing speciation rates. To be fair I actually also have a very hard time imagining how a 100x100 km grid should look like on the surface of the Earth? How could squares form a grid on a sphere ?

In short, a potentially promising paper that brings an interesting new resource to test an important question in biogeography, but with potential technical issues that make me concerned for the stability of the inference here.

Reviewer #2 (Remarks to the Author):

General Comments:
I found this to be an interesting manuscript that seeks to make an important contribution to our understanding of factors that cause the patterns of diversity we see in both the modern world and the paleo record. The data brought to bear on the questions of interest represent a very large and exciting contribution by themselves. The use of structural equation modeling to disentangle factors that explain speciation rates is central to their approach, and nicely conveys both hypotheses and results.

(1) The biggest difficulty I have with the current version of their manuscript relates to their inclusion of Figure 2, which combines uplift and eroded sites. This starts the paper off on the wrong foot and also exposes an additional problem that needs to be addressed (considered below). The authors find that the full data represented in Figure 2 are heterogeneous and when they break out uplift sites from erosion sites in Figures S2 and S3, they reveal what they really found and the basis for their summary conclusions. This order of presentation, and related issues, make the current version of the manuscript incoherent, in my view. The solution is simple. The analysis of the combined data set is worthy only of a mention in the methods section, not the inclusion of figures and tables summarizing the results. Those results are not the real story. In the world of causal modeling, the "real" story is one that is based on homogenous data. The result from analysis of a heterogeneous sample is a "false" story, not one you want to tell and give greater priority to.

(2) A second major issue has to do with the use of p-value based significance testing when in possession of a large sample size. Aside from the foundational problems with this approach, the practical matter arises at line 89-90, where the authors state that "Greater historical elevation changes not only directly promoted faster speciation, but also strongly influenced present-day elevation and subsequently temperature and speciation (Fig. 2)."

When we examine Figure 2, we see that the direct effect they are referring to has a standardized effect size of 0.044 for mammals and 0.038 for birds. These are by any scientific standard trivial effect sizes. Yes, the authors find them to be "statistically significant", but this is the precise reason quantitative scientists widely declare dichotomous significance testing inappropriate for applications such as this. Rather than belabor this point, I provide a link to article explaining the modern view of model selection and interpretation for models of the sort they are presenting (https://doi.org/10.3897/oneeco.5.e50452).

(3) Related to point #2, I do not find Figure 3 to be useful, given the use of color codes that are absolutely uninterpretable. You need to report numeric estimates for the total and indirect effects.

The upshot of the two main points raised here is that the authors need to focus the paper on the analyses of uplifted and eroded data up front, which will require major changes to the manuscript. They also need to set a priori limits for what they will declare to be a scientifically-relevant effect size. Normally, that value would be set at 0.01, with smaller values considered either "unimportant" or "of

lesser importance".

Reviewer #3 (Remarks to the Author):

The authors use case-effect methods to correlate global topographic uplift and temperature changes over the last 3myr with speciation rates in mammals and birds. The authors do a good job in communicating their findings. A few critical points, however, remain to be further explored before publication.

Major points:
Firstly, the authors should explore the uncertainties related to paleo-topographic estimations and temperature changes. These methods are known to contain large uncertainties and those are not clearly addressed in the manuscript. I recommend the inclusion of additional paleo reconstructions or considering effects of uncertainties on the statistical analysis. Secondly, although case-effect statistical methods are used, processes such as uplift and erosion are coupled, and should be further disentangled.
The authors found that in eroded areas, temperature has a stronger effect on speciation rate than elevation (line 121-122). It is hard to imagine an eroded area with a lower temperature than a surrounding site/cell with higher elevation. I find this conclusion premature, as elevation and temperature are coupled, as the authors highlight. Disentangling elevation, temperature and speciation rates might require local analysis of species pool, which may drastically influence species richness.

Minor points:
How did the authors define erosion (line 110)? As a decrease in elevation? This is somehow unclear and could benefit from another definition.
Did the authors group all cells with uplift and erosion or did they group them locally? E.g. for same geological systems?
Line 11. Consider replacing first and with "coma" or second and by "as well as".
Line 105. Remove first full stop before respectively.
Line 141. Remove first full stop ". (Fig. 4b).".
Figure 4. Color scheme of the figures were counterintuitive to me, decrease was blue and increase was red, could the authors change this?

Author Rebuttal to Initial comments

Reviewer #1:
This is a reasonably well-written and potentially very interesting analysis that adresses the hypothesis that active dynamics of mountain uplift has driven speciation globally. The key new thing they bring to the table is a map of elevation at high spatial resolution from 3 million years ago, allowing a high-resolution map of which grid cells have been uplifted or eroded away in the time since. This represents a nice resource to test a question that has otherwise been relatively intractable. Somewhat inexplicably, though, that map doesn't appear anywhere in the paper,

which I feel rather inhibits the ability to evaluate the analysis.

The authors use path analysis to evaluate the relative contributions of current values of elevation and temperature and the changes in those variables. That seems to me to be a relevant way to do it, and the relationships between the variables is realistically represented. This is one way of addressing the inherent lack of mutual independence in the contrasted predictors.
We are glad the Reviewer appreciates the work. Beyond the map, the "key new thing" we bring is a quantitative test (i.e. estimating the direction and magnitude) of how speciation is shaped by changes in both elevation and climate globally rather than just in mountains. This approach is possible by mapping speciation rates to global grids of both topographic and climate and separating interlinked processes within a path analysis framework. We have now added the map as Fig. S1 to highlight this new resource.

However, the big problem when doing this type of analysis is that uplift, of course, is associated with mountains, and a host of other mechanisms have associated mountains with increased species richness / speciation, incl climatic dynamics, tight packing of climate types, and complexity of geological substrates. Because I haven't seen the actual uplift map it's hard for me to tell whether their result simply is that you have more speciation in mountains, which is well known.
Added on lines 89-91:
"The relatively fine spatial resolution (100 x 100 km) of the paleo-relief model[28], allowed us to model changes worldwide rather than only in mountains as considered previously[8,9] (**Fig. S1**)."

Added on lines 317-319:
"We estimated the historical change in elevation as the difference in mean elevation per cell between the present and past (**Fig. S1**)."

Added on lines 355-358:
"**Figure S1. Global elevation changes since the mid-Piacenzian.** Pixels are at a 100 km × 100 km resolution and were calculated by subtracting past elevation in the mid-Piacenzian (~3 Ma) from the PRISM4 reconstruction from the ETOPO1 present-day elevation raster."

We'd also like to point out that while we certainly agree that "more speciation in mountains" is well known, the effects of change in elevation globally and its interplay with other abiotic variables has never been quantified as we do here.

Also, their method for evaluating specation rates, the DR metric, is not associated with a certain time scale (and has massive problems of phylogenetic autocorrelation which are very hard to deal with - and the same goes for bamm), thought they are explicitly looking at uplift over the last 3 mio years. I would think a metric of speciation rate explicitly in the time period was more relevant.

Tip rates estimated with model-free metrics like the DR statistic and model-based approaches like BAMM are both explicitly "conditional on past (usually recent) evolution history" (Title & Rabosky 2019, pg 822). Thus, the metrics are associated with recent speciation and precisely assume/model phylogenetic autocorrelation, which is how evolution works. Nonetheless, we have repeated our analyses only for the last 3 Ma and our conclusions remain unchanged.

Added on lines 286-289:
"Both of our speciation rates were conditioned on evolutionary history in the recent past. Specifically, rates estimated along the terminal branches of phylogenetic trees represent the waiting time in the present-day before a lineage will undergo another speciation event[54]."

Added on lines 182-184:
"Finally, our results were unchanged when we repeated our analyses using spatially explicit speciation rates estimated only over the period of our paleo-elevation data, i.e. last 3 Ma (Fig. S8)."

Added on lines 407-419:
"**Figure S8. Path analysis of hypothetical causal links among geologic and climatic factors and different grid-based speciation rates over the last 3 million years**. There were 762 and 1923 species of mammals and birds with an age <3 Myr, respectively. Including only these species in our analyses resulted in excluding 363 and 242 cells where all mammals and bird species were older, respectively. We then calculated the weighted mean λ_{BAMM} in a) uplifted (n = 3719 cells) and b) eroded (n = 10,528 cells for mammals and birds, respectively). As in our main analyses, we found that historic changes in speciation had comparable effects on speciation to current elevation (compare panel a) to Fig S5a and panel b) to Fig S6a). This result was unsurprising given the strong correlation between speciation rates. Weighted mean λ_{BAMM} for species younger than 3Myr was strongly positively correlated with rates calculated for all species reported in the main text ($\rho = 0.87$ and 0.93 for mammals and birds, respectively, $p < 0.001$ for both. Lines and colours as in Figure 2."

A theoretical concern is that they use temperature as a predictor of speciation rates, but that does not actually have a clear evolutionary interpretation, as far as I know. They argue that temperature might affect mutation rates, and thus the accumulation of genetic divergence among populations, but that hypothesis explicitly does not concern endotherms, which are the study subjects here.
There is a rich literature interpreting how temperature influences evolution of endotherms, specifically speciation rates. We have now tried to do a better job summarising this work.

Added on lines 42-63:
"Topographic changes also indirectly promote speciation because they modify climate, which itself affects speciation in at least four ways. First, warmer temperatures at lower elevations may

increase mutation rates, either directly through oxygen radical production[10] or faster metabolic rates that increase DNA synthesis[11], and shorten generation times, thereby increasing the likelihood of speciation[12,13] but see[14,15]. Although endotherms may escape these effects by buffering their body temperatures[16], warmer temperatures will reduce the costs of maintaining metabolic rates[17]. Lower thermoregulatory costs can release energy that enables large population sizes and promotes speciation by allowing more diverse ecological niches to be occupied[18,19]. Second, species interactions, such as competition, predation, and mutualism are a constant source of macroevolutionary change – so called Red Queen effects – and these are expected to be more intense at warmer temperatures[20]. Third, species tend to have wider thermal niches in colder climates because they experience greater temperature variation[21]. This broader niche can reduce opportunities for reproductive isolation and thus speciation if populations are consequently more widespread[20,22]. There may also be greater physiological costs of wider temperature adaptation that restricts the number of species that can employ this strategy[18]. Finally, greater climatic instability arising from topographic change may have promoted rapid and repeated ecological speciation by creating novel niches and increased ecological opportunity[16,23]. This relationship between climatic instability and speciation may, however, be non-linear, since higher speciation rates have also been linked with areas of high climatic stability like Pleistocene refugia[24]."

With regards to their analysis I don't understand why they reapply a 1-degree grid net to a 100x100 km equal area grid. The data is on 1-degree, any reprojection at the same resolution must necessarily throw away great amounts of data. I don't immediately see how different sizes towards the poles of the 1-deg should be a problem for analyzing speciation rates. To be fair I actually also have a very hard time imagining how a 100x100 km grid should look like on the surface of the Earth? How could squares form a grid on a sphere ?
No data are lost by reprojection so there is no need to make changes to the text or analyses here. Our intention is to standardise grid cells to control for differences in speciation simply due to differences in study area. A larger study area, could for example, include more species and more estimates so we want to ensure this isn't biasing our results. Re-projecting data is standard in these sorts of geospatial analyses and the grid is shaped around the sphere to accommodate this (*please see image below of grid on a sphere*).

Example of square grid network on Earth. ►

[REDACTED]

In short, a potentially promising paper that brings an interesting new resource to test an important question in biogeography, but with potential technical issues that make me concerned for the stability of the inference here.
Again, we thank the Reviewer for their constructive feedback, which has improved the MS. We hope that our response resolves their technical concerns.

Reviewer #2:
General Comments:
I found this to be an interesting manuscript that seeks to make an important contribution to our understanding of factors that cause the patterns of diversity we see in both the modern world and the paleo record. The data brought to bear on the questions of interest represent a very large and exciting contribution by themselves. The use of structural equation modeling to disentangle factors that explain speciation rates is central to their approach, and nicely conveys both hypotheses and results.
We thank the Reviewer for appreciating the value and excitement of the work.

(1) The biggest difficulty I have with the current version of their manuscript relates to their inclusion of Figure 2, which combines uplift and eroded sites. This starts the paper off on the wrong foot and also exposes an additional problem that needs to be addressed (considered below). The authors find that the full data represented in Figure 2 are heterogeneous and when they break out uplift sites from erosion sites in Figures S2 and S3, they reveal what they really found and the basis for their summary conclusions. This order of presentation, and related issues, make the current version of the manuscript incoherent, in my view. **The solution is simple** *[emphasis added]*. The analysis of the combined data set is worthy only of a mention in the methods section, not the inclusion of figures and tables summarizing the results. Those results are not the real story. In the world of causal modeling, the "real" story is one that is based on homogenous data. The result from analysis of a heterogeneous sample is a "false" story, not one you want to tell and give greater priority to.
We entirely agree and debated this decision between ourselves. We have now done exactly what the Reviewer suggests – focus on the separate analysis of uplift and erosion sites (i.e. the homogenous data) and then briefly mention the combined dataset to illustrate the importance of separately considering uplift and erosion processes in all future studies.

Changed on lines 79-89:
"Here we quantified how much of the present-day spatial variation in speciation rates of mammals and birds was explained by climate and geology and their changes since the Plio-Pleistocene approximately 3 million years ago (Ma). … As elevation changes can arise from both the gain and loss of topography, which we respectively termed uplift and erosion, we determined if these two opposing processes had contrasting effects on speciation."

Changed on lines 99-152:
"**Results**
We found that speciation rates increased most in areas with the greatest increases in elevation from the Plio-Pleistocene (Fig. 2). We separately analysed 3,780 and 10,884 100-km grid cells that underwent uplift and erosion, respectively, since the mid-Piacenzian. The positive effect of elevation gain on speciation rates was stronger than that of present-day elevation and outweighed the effects of both temperature in the present-day and its historical change (Fig. 3a).

Consequently, speciation rates increased by a mean of 11% (95% confidence interval, CI: 9 to 13%) and 10% (95% CI: 8 to 12%) in mammals and birds, respectively, for a 1 standard deviation (SD) gain in elevation of 195 m above the mean change in uplift cells of 144 m. Both the direct and indirect effects of elevation change contributed to these results. Greater historical uplift strongly influenced present-day elevation and subsequently temperature and speciation (Fig. 2). Overall, in uplift areas, the total effects of elevation on speciation rates were larger than the effects of temperature when aggregating both these direct and indirect effects (Fig. 3c). Historical changes in temperature and elevation were also much stronger drivers of speciation in uplifted areas than the present-day values of these variables (Fig. 3e).

Erosion had contrasting effects to those of uplift. Although elevation change causing erosion promoted speciation (Fig. S3), it was directly correlated with 5.5- and 11.9-times lower speciation rates than uplift for mammals and birds, respectively (Figs 3a, b). For example, a 1 SD reduction in elevation beneath the mean temporal change of -115 m in eroded cells increased speciation only by a mean of 2% (95% CI: 1 to 3%) and 1% (95% CI: <1% to 2%) in mammals and birds, respectively. Instead, temperature, primarily present-day values, had the strongest effect on speciation rates in eroded grid cells (Fig. 3b,d). Historical changes of both elevation and temperature had weaker effects than present-day values (Fig. 3f).

The strong effect of topographic change on speciation rates diminished when we analysed all areas together irrespective of whether they underwent uplift or erosion, highlighting the importance of separating these processes (Fig. 4a). …"

(2) A second major issue has to do with the use of p-value based significance testing when in possession of a large sample size. Aside from the foundational problems with this approach, the practical matter arises at line 89-90, where the authors state that "Greater historical elevation changes not only directly promoted faster speciation, but also strongly influenced present-day elevation and subsequently temperature and speciation (Fig. 2)."

When we examine Figure 2, we see that the direct effect they are referring to has a standardized effect size of 0.044 for mammals and 0.038 for birds. These are by any scientific standard trivial effect sizes. Yes, the authors find them to be "statistically significant", but this is the precise reason quantitative scientists widely declare dichotomous significance testing inappropriate for applications such as this. Rather than belabor this point, I provide a link to article explaining the modern view of model selection and interpretation for models of the sort they are presenting (https://doi.org/10.3897/oneeco.5.e50452).

We entirely agree with the Reviewer about the problems with p-values and dichotomous significance testing and are not advocating that the effects they highlight in their comment are necessarily strong. However, the question is what are "trivial effect sizes"?

There is no clearly accepted threshold of trivial/non-trivial effects. Instead we must look at model predictions and ask what effects are ***biologically*** relevant. We argue that we should consider changes in speciation rates of at least 5% for every SD change in a predictor to be

biologically relevant, especially given the timescales and processes we consider here. As our focal response variable (speciation rate) was log-transformed, a 5% change over the observed speciation rates corresponds to a standardised effect of log(1.05) = 0.049. This effect size can be derived by calculating the relative change in speciation required to correspond to a 5% difference, i.e. $(e^{x+y} – e^{x})/e^{x} = 0.05$, where x is any speciation rate on the log-scale and y is the effect size required to increase it by 5%; this equation solves to log(1.05).

Therefore, we have taken two actions to address the Reviewer's comment:

1) Only reported effect sizes >0.049 in figures, i.e. corresponding with a 5% change in speciation for every 1 SD change in a predictor.

Added on lines 121-125:
"Positive, negative and non-statistically significant (p-value > 0.05) relationships are indicated by blue, red and grey arrows, respectively. The width of the arrows is proportional to the standardised effect size shown by adjacent numbers. Numbers are only reported for those effect sizes that cause at least a 5% change in a response for each standard deviation change in the corresponding predictor."

Added on lines 145-148:
"Values are standardised effect sizes and are only shown where they cause at least a 5% change in a response for each standard deviation change in the corresponding predictor."

2) Added additional text to interpret biological effects.

Added on lines 105-108:
"Consequently, speciation rates increased by a mean of 11% (95% confidence interval, CI: 9 to 13%) and 10% (95% CI: 8 to 12%) in mammals and birds, respectively, for a 1 standard deviation (SD) gain in elevation of 195 m above the mean change in uplift cells of 144 m."

Added on lines 132-134:
"For example, a 1 SD reduction in elevation beneath the mean temporal change of -115 m in eroded cells increased speciation only by a mean of 2% (95% CI: 1 to 3%) and 1% (95% CI: <1% to 2%) in mammals and birds, respectively."

(3) Related to point #2, I do not find Figure 3 to be useful, given the use of color codes that are absolutely uninterpretable. You need to report numeric estimates for the total and indirect effects.

As requested, we have added numeric estimates for statistically significant total and indirect effects to Fig. 3 and Fig. 4.

Added on lines 145-148:

"Values are standardised effect sizes and are only shown where they cause at least a 5% change in a response for each standard deviation change in the corresponding predictor."

The upshot of the two main points raised here is that the authors need to focus the paper on the analyses of uplifted and eroded data up front, which will require major changes to the manuscript. They also need to set a priori limits for what they will declare to be a scientifically-relevant effect size. Normally, that value would be set at 0.01, with smaller values considered either "unimportant" or "of lesser importance".
As described above in response to points (1) and (2), we have focused the paper upfront on the analyses of uplift and eroded data and set a priori limits for scientifically relevant effect sizes. The exact changes to the text are given above and so are not reported here. Like the Reviewer says, the upshot is that these criticisms only require changes to the text and not our analyses or conclusions, all of which still hold. Thank you again for your helpful comments!

Reviewer #3:
The authors use case-effect methods to correlate global topographic uplift and temperature changes over the last 3myr with speciation rates in mammals and birds. The authors do a good job in communicating their findings. A few critical points, however, remain to be further explored before publication.
We thank the Reviewer for their positive comments on the communication of our findings.

Major points:
Firstly, the authors should explore the uncertainties related to paleo-topographic estimations and temperature changes. These methods are known to contain large uncertainties and those are not clearly addressed in the manuscript. I recommend the inclusion of additional paleo reconstructions or considering effects of uncertainties on the statistical analysis.
We certainly agree that there are uncertainties in the palaeo-reconstructions. Unfortunately, no quantitative estimates exist for these models. The only thing we could possibly do is add random noise to the palaeo-reconstructions and re-run our statistical analyses, but it isn't even clear what level of variation would be reasonable (e.g. standard deviation = 5%, 10%, 30% of mean elevation?) or how this might even scale with mean values (e.g. linearly with mean elevation or perhaps it is constant error?). We think that it is best to avoid carrying out such analyses to create false confidence in the uncertainty analyses. Instead, we have added text to the Discussion that clearly addresses these challenges and the potential future advances in this area.

Added on lines 239-244:
"One limitation is that we were unable to estimate the uncertainty related to the paleo-reconstructions as quantitative estimates do not yet exist. As novel paleo-altimetric methods are further refined and more precise reconstructions of past elevation are generated[40,41], the association between speciation and geological changes can be improved and assessed in older epochs than the Plio-Pleistocene."

Secondly, although case-effect statistical methods are used, processes such as uplift and erosion are coupled, and should be further disentangled.
We entirely agree and debated this decision between ourselves. We have now restructured our paper around disentangling uplift and erosion and only briefly mention the combined dataset at the end of the paper.

Changed on lines 79-89:
"Here we quantified how much of the present-day spatial variation in speciation rates of mammals and birds was explained by climate and geology and their changes since the Plio-Pleistocene approximately 3 million years ago (Ma). … As elevation changes can arise from both the gain and loss of topography, which we respectively termed uplift and erosion, we determined if these two opposing processes had contrasting effects on speciation."

Changed on lines 99-152:
"**Results**
We found that speciation rates increased most in areas with the greatest increases in elevation from the Plio-Pleistocene (Fig. 2). We separately analysed 3,780 and 10,884 100-km grid cells that underwent uplift and erosion, respectively, since the mid-Piacenzian. The positive effect of elevation gain on speciation rates was stronger than that of present-day elevation and outweighed the effects of both temperature in the present-day and its historical change (Fig. 3a). Consequently, speciation rates increased by a mean of 11% (95% confidence interval, CI: 9 to 13%) and 10% (95% CI: 8 to 12%) in mammals and birds, respectively, for a 1 standard deviation (SD) gain in elevation of 195 m above the mean change in uplift cells of 144 m. Both the direct and indirect effects of elevation change contributed to these results. Greater historical uplift strongly influenced present-day elevation and subsequently temperature and speciation (Fig. 2). Overall, in uplift areas, the total effects of elevation on speciation rates were larger than the effects of temperature when aggregating both these direct and indirect effects (Fig. 3c). Historical changes in temperature and elevation were also much stronger drivers of speciation in uplifted areas than the present-day values of these variables (Fig. 3e).

Erosion had contrasting effects to those of uplift. Although elevation change causing erosion promoted speciation (Fig. S3), it was directly correlated with 5.5- and 11.9-times lower speciation rates than uplift for mammals and birds, respectively (Figs 3a, b). For example, a 1 SD reduction in elevation beneath the mean temporal change of -115 m in eroded cells increased speciation only by a mean of 2% (95% CI: 1 to 3%) and 1% (95% CI: <1% to 2%) in mammals and birds, respectively. Instead, temperature, primarily present-day values, had the strongest effect on speciation rates in eroded grid cells (Fig. 3b,d). Historical changes of both elevation and temperature had weaker effects than present-day values (Fig. 3f).

The strong effect of topographic change on speciation rates diminished when we analysed all areas together irrespective of whether they underwent uplift or erosion, highlighting the importance of separating these processes (Fig. 4a). …"

The authors found that in eroded areas, temperature has a stronger effect on speciation rate than elevation (line 121-122). It is hard to imagine an eroded area with a lower temperature than a surrounding site/cell with higher elevation. I find this conclusion premature, as elevation and temperature are coupled, as the authors highlight.

Our analysis is specifically designed to disentangle the effects of elevation and temperature on speciation rates. The Reviewer is certainly correct that eroded areas are unlikely to have lower temperatures than surrounding cells with higher elevation, but that doesn't mean that temperature is necessarily the causal agent for this effect. Separately analysing eroded and uplift areas with case-effect statistical methods, as suggested in response to the Reviewer's second major comment, allows us to address this concern. We have now restructured the entire Results around

disentangling uplift and erosion. We provide the full changes to the text above in response to the second major comment, so do not repeat them here.

Disentangling elevation, temperature and speciation rates might require local analysis of species pool, which may drastically influence species richness.
We are unclear from this comment why the local species pool is relevant to test the effects of elevation and temperature on speciation. Speciation rates may certainly differ in some places compared to others because of the species that are locally present, but that is precisely the point of analysing species-specific speciation rates. If speciation rates differ, it is because of the species that are present, i.e. differences in their speciation rates, and we seek to understand how these differences relate to elevation and temperature. We have clarified this point in the text.
Added on lines 298-301:
"We mapped speciation rates by overlaying species ranges with a grid of 100 km by 100 km cells. This approach allowed us to account for variation in local species pools among grid cells. Following past studies[14,29], we then computed the speciation rate for individual grid cells."

Minor points:
How did the authors define erosion (line 110)? As a decrease in elevation? This is somehow unclear and could benefit from another definition.
Changed on lines 86-89:
"As elevation changes can arise from both the gain and loss of topography, which we respectively termed uplift and erosion, we determined if these two opposing processes had contrasting effects on speciation."

Did the authors group all cells with uplift and erosion or did they group them locally? E.g. for same geological systems?
We did not group uplift and erosion as this analysis is spatially explicit for grid cells that are classified as having undergone either uplift or erosion. We believe this comment stemmed from this sentence:

> "The strong effect of topographic change on speciation rates only diminished when we grouped together areas with uplift and erosion (Fig. 4a)."

Therefore, we have reworded the text to clarify that we also considered topographic change as a whole, i.e. irrespective of whether grid cells underwent uplift or erosion, and there was no grouping.

Changed on lines 150-152:
"The strong effect of topographic change on speciation rates diminished when we analysed all areas together irrespective of whether they underwent uplift or erosion, highlighting the importance of separating these processes (Fig. 4a)."

Line 11. Consider replacing first and with "coma" or second and by "as well as".
Changed on lines 10-11:
"Topographic change shapes the evolution of biodiversity by influencing both habitat connectivity and diversity as well as abiotic factors like climate."

Line 105. Remove first full stop before respectively. *Removed.*
Line 141. Remove first full stop ". (Fig. 4b).". Thanks for spotting! *Removed.*
Figure 4. Color scheme of the figures were counterintuitive to me, decrease was blue and increase was red, could the authors change this? We have inverted the colour scheme throughout.

Thank you again for your helpful comments that have helped to improve the MS!

Decision Letter, first revision:

28th June 2021

Dear Andrew,

Thank you for submitting your revised manuscript "Global topographic uplift has elevated speciation in mammals and birds over the last 3 million years" (NATECOLEVOL-210112759A). It has now been seen again by the original reviewers and their comments are below. Unfortunately, Reviewer #1 could not review again but we asked another researcher with similar expertise (Reviewer #4) to check your responses to their comments. The reviewers find that the paper has improved in revision, and therefore we'll be happy in principle to publish it in Nature Ecology & Evolution, pending minor revisions to comply with our editorial and formatting guidelines.

If the current version of your manuscript is in a PDF format, please email us a copy of the file in an editable format (Microsoft Word or LaTex)-- we can not proceed with PDFs at this stage.

We are now performing detailed checks on your paper and will send you a checklist detailing our editorial and formatting requirements in about a week. Please do not upload the final materials and make any revisions until you receive this additional information from us.

Thank you again for your interest in Nature Ecology & Evolution. Please do not hesitate to contact me if you have any questions.

[REDACTED]

Reviewer #2 (Remarks to the Author):

I am very pleased that the reviewers found the suggestions useful. This is a very exciting paper that addresses questions of broad interest, but of course encounters numerous technical challenges along the way. I feel the authors have done a nice job addressing those technical challenges and presenting the findings clearly and convincingly.
Jim Grace

Reviewer #3 signs off with no further comments to the authors.

Reviewer #4 (Remarks to the Author):

The authors have addressed the concerns of reviewer number 1 sufficiently.
The authors have included the recommended map in the supplement. However, uplift appears to be presented as negative elevational change, which seems counter intuitive. The shape of the map appears to be a bit distorted by compression. The analysis has been repeated for a different timeframe which did not change the conclusions. They have now explained how temperature can influence speciation. The reprojection of grid cells has been explained and no data was lost.

Our ref: NATECOLEVOL-210112759A

30th June 2021

Dear Dr. Tanentzap,

Thank you for your patience as we've prepared the guidelines for final submission of your Nature Ecology & Evolution manuscript, "Global topographic uplift has elevated speciation in mammals and birds over the last 3 million years" (NATECOLEVOL-210112759A). Please carefully follow the step-by-step instructions provided in the attached file, and add a response in each row of the table to indicate the changes that you have made. Please also check and comment on any additional marked-up edits we have proposed within the text. Ensuring that each point is addressed will help to ensure that your revised manuscript can be swiftly handed over to our production team.

We would like to start working on your revised paper, with all of the requested files and forms, as soon as possible (preferably within two weeks). Please get in contact with us immediately if you anticipate it taking more than two weeks to submit these revised files.

When you upload your final materials, please include a point-by-point response to any remaining reviewer comments.

If you have not done so already, please alert us to any related manuscripts from your group that are under consideration or in press at other journals, or are being written up for submission to other journals (see: https://www.nature.com/nature-research/editorial-policies/plagiarism#policy-on-duplicate-publication for details).

In recognition of the time and expertise our reviewers provide to Nature Ecology & Evolution's editorial process, we would like to formally acknowledge their contribution to the external peer review of your manuscript entitled "Global topographic uplift has elevated speciation in mammals and birds over the last 3 million years". For those reviewers who give their assent, we will be publishing their names alongside the published article.

Nature Ecology & Evolution offers a Transparent Peer Review option for new original research manuscripts submitted after December 1st, 2019. As part of this initiative, we encourage our authors to support increased transparency into the peer review process by agreeing to have the reviewer comments, author rebuttal letters, and editorial decision letters published as a Supplementary item. When you submit your final files please clearly state in your cover letter whether or not you would like to participate in this initiative. Please note that failure to state your preference will result in delays in accepting your manuscript for publication.

Cover suggestions

As you prepare your final files we encourage you to consider whether you have any images or illustrations that may be appropriate for use on the cover of Nature Ecology & Evolution.

Covers should be both aesthetically appealing and scientifically relevant, and should be supplied at the best quality available. Due to the prominence of these images, we do not generally select images featuring faces, children, text, graphs, schematic drawings, or collages on our covers.

We accept TIFF, JPEG, PNG or PSD file formats (a layered PSD file would be ideal), and the image should be at least 300ppi resolution (preferably 600-1200 ppi), in CMYK colour mode.

If your image is selected, we may also use it on the journal website as a banner image, and may need to make artistic alterations to fit our journal style.

Please submit your suggestions, clearly labeled, along with your final files. We'll be in touch if more information is needed.

Nature Ecology & Evolution has now transitioned to a unified Rights Collection system which will allow our Author Services team to quickly and easily collect the rights and permissions required to publish your work. Approximately 10 days after your paper is formally accepted, you will receive an email in providing you with a link to complete the grant of rights. If your paper is eligible for Open Access, our Author Services team will also be in touch regarding any additional information that may be required to arrange payment for your article.

Please note that <i>Nature Ecology & Evolution</i> is a Transformative Journal (TJ). Authors may publish their research with us through the traditional subscription access route or make their paper

immediately open access through payment of an article-processing charge (APC). Authors will not be required to make a final decision about access to their article until it has been accepted. Find out more about Transformative Journals

<B>Authors may need to take specific actions to achieve compliance with funder and institutional open access mandates. For submissions from January 2021, if your research is supported by a funder that requires immediate open access (e.g. according to Plan S principles) then you should select the gold OA route, and we will direct you to the compliant route where possible. For authors selecting the subscription publication route our standard licensing terms will need to be accepted, including our self-archiving policies. Those standard licensing terms will supersede any other terms that the author or any third party may assert apply to any version of the manuscript.

Please note that you will not receive your proofs until the publishing agreement has been received through our system.

For information regarding our different publishing models please see our Transformative Journals page. If you have any questions about costs, Open Access requirements, or our legal forms, please contact ASJournals@springernature.com.

Please use the following link for uploading these materials:
[REDACTED]

If you have any further questions, please feel free to contact me.

[REDACTED]

Reviewer #2:
Remarks to the Author:
I am very pleased that the reviewers found the suggestions useful. This is a very exciting paper that addresses questions of broad interest, but of course encounters numerous technical challenges along the way. I feel the authors have done a nice job addressing those technical challenges and presenting the findings clearly and convincingly.
Jim Grace

Reviewer #3:
None

Reviewer #4:
Remarks to the Author:
The authors have addressed the concerns of reviewer number 1 sufficiently.
The authors have included the recommended map in the supplement. However, uplift appears to be presented as negative elevational change, which seems counter intuitive. The shape of the map appears to be a bit distorted by compression. The analysis has been repeated for a different timeframe which did not change the conclusions. They have now explained how temperature can influence speciation. The reprojection of grid cells has been explained and no data was lost.

Author Rebuttal, first revision:

Reviewer #2:
I am very pleased that the reviewers found the suggestions useful. This is a very exciting paper that addresses questions of broad interest, but of course encounters numerous technical challenges along the way. I feel the authors have done a nice job addressing those technical challenges and presenting the findings clearly and convincingly.
Jim Grace
Thank you very much for the positive feedback!

Reviewer #3:
None
No response required.

Reviewer #4:
The authors have addressed the concerns of reviewer number 1 sufficiently.
Thank you!

The authors have included the recommended map in the supplement. However, uplift appears to be presented as negative elevational change, which seems counter intuitive.
As explained in the previous response, uplift is presented in red as positive elevational change. We have now made this more explicit in the figure legend.

The shape of the map appears to be a bit distorted by compression.
The shape of the map appears distorted because we have used an "equal-areas projection" to show grid cells, which are the same size on Earth, as the same size on the map. Because of the curvature of the Earth, this projection makes northern areas look larger than we typically see them on a sphere and gives the impression of "distortion". We have widened the plot to make the grid cells appear more square-like and minimise the impression of "compression".

The analysis has been repeated for a different timeframe which did not change the conclusions. They have now explained how temperature can influence speciation. The reprojection of grid cells has been explained and no data was lost.
No specific point is raised in these comments, so no further response is required.

Final Decision Letter:

26th July 2021

Dear Dr Tanentzap,

We are pleased to inform you that your Article entitled "Global topographic uplift has elevated speciation in mammals and birds over the last 3 million years", has now been accepted for publication in Nature Ecology & Evolution.

Before your manuscript is typeset, we will edit the text to ensure it is intelligible to our wide readership and conforms to house style. We look particularly carefully at the titles of all papers to ensure that they are relatively brief and understandable.

The subeditor may send you the edited text for your approval. Once your manuscript is typeset you will receive a link to your electronic proof via email, with a request to make any corrections within 48 hours. If you have queries at any point during the production process then please contact the production team at rjsproduction@springernature.com. Once your paper has been scheduled for online publication, the Nature press office will be in touch to confirm the details.

Acceptance of your manuscript is conditional on all authors' agreement with our publication policies (see www.nature.com/authors/policies/index.html). In particular your manuscript must not be published elsewhere and there must be no announcement of the work to any media outlet until the publication date (the day on which it is uploaded onto our web site).

Please note that <i>Nature Ecology & Evolution</i> is a Transformative Journal (TJ). Authors may publish their research with us through the traditional subscription access route or make their paper immediately open access through payment of an article-processing charge (APC). Authors will not be required to make a final decision about access to their article until it has been accepted. Find out more about Transformative Journals

<B>Authors may need to take specific actions to achieve compliance with funder and institutional open access mandates. For submissions from January 2021, if your research is supported by a funder that requires immediate open access (e.g. according to Plan S principles) then you should select the gold OA route, and we will direct you to the compliant route where possible. For authors selecting the subscription publication route our standard licensing terms will need to be accepted, including our <a href="https://www.springernature.com/gp/open-

research/policies/journal-policies">self-archiving policies. Those standard licensing terms will supersede any other terms that the author or any third party may assert apply to any version of the manuscript.

In approximately 10 business days you will receive an email with a link to choose the appropriate publishing options for your paper and our Author Services team will be in touch regarding any additional information that may be required.

You will not receive your proofs until the publishing agreement has been received through our system.

If you have any questions about our publishing options, costs, Open Access requirements, or our legal forms, please contact ASJournals@springernature.com

An online order form for reprints of your paper is available at https://www.nature.com/reprints/author-reprints.html. All co-authors, authors' institutions and authors' funding agencies can order reprints using the form appropriate to their geographical region.

We welcome the submission of potential cover material (including a short caption of around 40 words) related to your manuscript; suggestions should be sent to Nature Ecology & Evolution as electronic files (the image should be 300 dpi at 210 x 297 mm in either TIFF or JPEG format). Please note that such pictures should be selected more for their aesthetic appeal than for their scientific content, and that colour images work better than black and white or grayscale images. Please do not try to design a cover with the Nature Ecology & Evolution logo etc., and please do not submit composites of images related to your work. I am sure you will understand that we cannot make any promise as to whether any of your suggestions might be selected for the cover of the journal.

You can now use a single sign-on for all your accounts, view the status of all your manuscript submissions and reviews, access usage statistics for your published articles and download a record of your refereeing activity for the Nature journals.

To assist our authors in disseminating their research to the broader community, our SharedIt initiative provides you with a unique shareable link that will allow anyone (with or without a subscription) to read the published article. Recipients of the link with a subscription will also be able to download and print the PDF.

You can generate the link yourself when you receive your article DOI by entering it here: http://authors.springernature.com/share<a>.

[REDACTED]

P.S. Click on the following link if you would like to recommend Nature Ecology & Evolution to your librarian http://www.nature.com/subscriptions/recommend.html#forms

** Visit the Springer Nature Editorial and Publishing website at <a href="http://editorial-

jobs.springernature.com?utm_source=ejP_NEcoE_email&utm_medium=ejP_NEcoE_email&utm_campaign=ejp_NEcoE">www.springernature.com/editorial-and-publishing-jobs for more information about our career opportunities. If you have any questions please click here.**
